# The dynamic transcriptome during maturation of biofilms formed by methicillin-resistant *Staphylococcus aureus*

**DOI:** 10.3389/fmicb.2022.882346

**Published:** 2022-07-28

**Authors:** Jelle Vlaeminck, Qiang Lin, Basil Britto Xavier, Sarah De Backer, Matilda Berkell, Henri De Greve, Jean-Pierre Hernalsteens, Samir Kumar-Singh, Herman Goossens, Surbhi Malhotra-Kumar

**Affiliations:** ^1^Laboratory of Medical Microbiology, Vaccine and Infectious Disease Institute, University of Antwerp, Antwerp, Belgium; ^2^Molecular Pathology Group, Laboratory of Cell Biology & Histology, University of Antwerp, Antwerp, Belgium; ^3^VIB-VUB Center for Structural Biology, Vrije Universiteit Brussel, Brussels, Belgium; ^4^Structural Biology Brussels, Vrije Universiteit Brussel, Brussels, Belgium; ^5^Department of Biology, Vrije Universiteit Brussel, Brussels, Belgium

**Keywords:** biofilm, MRSA, transcriptome, clumping factor A, *Staphylococcus aureus*

## Abstract

**Background:**

Methicillin-resistant *Staphylococcus aureus* (MRSA), a leading cause of chronic infections, forms prolific biofilms which afford an escape route from antibiotic treatment and host immunity. However, MRSA clones are genetically diverse, and mechanisms underlying biofilm formation remain under-studied. Such studies form the basis for developing targeted therapeutics. Here, we studied the temporal changes in the biofilm transcriptome of three pandemic MRSA clones: USA300, HEMRSA-15, and ST239.

**Methods:**

Biofilm formation was assessed using a static model with one representative strain per clone. Total RNA was extracted from biofilm and planktonic cultures after 24, 48, and 72 h of growth, followed by rRNA depletion and sequencing (Illumina Inc., San Diego, CA, United States, NextSeq500, v2, 1 × 75 bp). Differentially expressed gene (DEG) analysis between phenotypes and among early (24 h), intermediate (48 h), and late (72 h) stages of biofilms was performed together with *in silico* co-expression network construction and compared between clones. To understand the influence of SCC*mec* and ACME on biofilm formation, isogenic mutants containing deletions of the entire elements or of single genes therein were constructed in USA300.

**Results:**

Genes involved in primarily core genome-encoded KEGG pathways (transporters and others) were upregulated in 24-h biofilm culture compared to 24-h planktonic culture. However, the number of affected pathways in the ST239 24 h biofilm (*n* = 11) was remarkably lower than that in USA300/EMRSA-15 biofilms (USA300: *n* = 27, HEMRSA-15: *n* = 58). The *clfA* gene, which encodes clumping factor A, was the single common DEG identified across the three clones in 24-h biofilm culture (2.2- to 2.66-fold). In intermediate (48 h) and late (72 h) stages of biofilms, decreased expression of central metabolic and fermentative pathways (glycolysis/gluconeogenesis, fatty acid biosynthesis), indicating a shift to anaerobic conditions, was already evident in USA300 and HEMRSA-15 in 48-h biofilm cultures; ST239 showed a similar profile at 72 h. Last, SCC*mec*+ACME deletion and *opp3D* disruption negatively affected USA300 biofilm formation.

**Conclusion:**

Our data show striking differences in gene expression during biofilm formation by three of the most important pandemic MRSA clones, USA300, HEMRSA-15, and ST239. The *clfA* gene was the only significantly upregulated gene across all three strains in 24-h biofilm cultures and exemplifies an important target to disrupt early biofilms. Furthermore, our data indicate a critical role for arginine catabolism pathways in early biofilm formation.

## Introduction

Microbial biofilms represent a critical determinant of human chronic bacterial infections. Bacterial biofilms involve a genetically coordinated sequence of events, including initial surface attachment, microcolony formation, and bacterial community expansion. This leads to a complex and structured architecture protecting bacteria from host defense mechanisms and killing by antimicrobials. Among the most clinically significant bacterial pathogens is *Staphylococcus aureus*, a leading cause of nosocomial and community-acquired infections (Archer et al., 2011). Considerable research efforts have been directed toward understanding the mechanisms of staphylococcal biofilm formation. Much of this research has focused on bacterial mediators (Dunman et al., 2001), environmental effectors (Kavanaugh and Horswill, 2016; Kim et al., 2016), and global changes that occur during biofilm development (Beenken et al., 2004; Resch et al., 2005, 2006). Transcriptional profiling studies of *S. aureus* biofilms have shown that bacteria grow micro-aerobically or anaerobically relative to planktonic cultures (Beenken et al., 2004; Resch et al., 2005, 2006). This is exemplified by a switch from aerobic energy production to fermentative processes such as acetoin metabolism, a general downregulation of active cell processes, such as protein, DNA, and cell wall biosynthesis, and increased expression of genes involved in arginine deiminase and urease pathways. Here, cells can gain energy in the form of ATP from the conversion of arginine to citrulline (Cunin et al., 1986). Also, the products of proteins encoded by the arginine deiminase gene cluster feed into the urea cycle and thereby lead to the generation of ammonia and/or urea to counteract the low-pH condition caused by the production of lactic acid and formic acid under anaerobic conditions. This switch from aerobic respiration to anaerobic fermentation has also been reported to be relevant for antibiotic resistance (Martínez and Rojo, 2011), transcription of virulence genes (Fuchs et al., 2007), and the expression of extracellular polysaccharides involved in cell-to-cell adhesion and biofilm formation (Cramton et al., 2001).

Methicillin-resistant *S. aureus* (MRSA) is a multidrug resistant variant of *S. aureus* (Lee et al., 2018), which developed from susceptible *S. aureus* clones by the uptake of *mecA*, encoding methicillin resistance, on the mobile staphylococcal cassette chromosome *mec* (SCC*mec*) element *via* horizontal gene transfer (Ito et al., 1999). Since their first isolation in the 1960s, MRSA clones, such as USA300, have become pandemic and a major cause of both hospital- (HA) and community-acquired (CA) infections as high levels of adaptability and rapid evolvement of antimicrobial resistance typify these clones (Center for Disease Dynamics: Economics & Policy, 2018). Several MRSA lineages have been previously described as prolific biofilm formers with great clinical implications because of the density of biofilm, enhancing antibiotic recalcitrance, immune evasion, and horizontal gene transfer rates (Costerton et al., 1999; Sørensen et al., 2005; Craft et al., 2019). In *S. aureus*, biofilm is mainly formed by *icaADBC* mechanisms, but importantly, biofilm formation in MRSA has been shown to differ considerably from that in MSSA strains, with the former forming more proteinaceous biofilms, independent of the *icaADBC* mechanism that prevails in MSSA biofilms (O’Neill et al., 2007). Similar results were obtained when *mecA* was artificially introduced in MSSA (Pozzi et al., 2012). Moreover, the acquisition of another element named arginine catabolic mobile element (ACME) has been suggested to also enhance biofilm formation in MRSA USA300 (Planet et al., 2013; Vanhommerig et al., 2014).

To gain deeper insights into biofilm formation across genetically diverse MRSA clones, three prolific biofilm-forming MRSA strains (UAS391, HEMRSA-15, and ST239-16), belonging to pandemic clonal lineages USA300, HEMRSA-15, and the Hungarian/Brazilian ST239 clone, respectively, were utilized in this study (Vanhommerig et al., 2014). These strains were chosen because they represent MRSA phenotypes from distinct genetic backgrounds [sequence type (ST)8, ST22, and ST239, respectively] that are common causes of biofilm-associated infections such as chronic wounds and device-associated infections globally (Boudet et al., 2021; Cascioferro et al., 2021). We studied differential gene expression over time in biofilm and planktonic phenotypes demonstrated by the three representative strains. The role of mobile genetic elements, SCC*mec* and/or ACME, in biofilm formation by USA300 was also investigated.

## Materials and methods

### Bacterial isolates

In order to study the common and varying mechanisms of biofilm formation in MRSA clones, three MRSA strains representing three pandemic MRSA clones were used: (i) UAS391 [USA300, PVL+/ACME+, abscess/wound isolate, 2591 coding sequences (CDS); mobile content: 5.12%] belonging to ST8, harboring SCC*mec* type IV, previously identified as a prolific biofilm former; (ii) HEMRSA-15 (EMRSA-15; unknown source, 2562 CDS; mobile content: 7.75%) belonging to ST22, harboring SCC*mec* type IV; and (iii) ST239-16 (Hungarian/Brazilian ST239, respiratory tract isolate, 2785 CDS, mobile content: 12.18%) belonging to ST239, harboring SCC*mec* type III. These strains have been shown previously by us to have strikingly different biofilm phenotypes in terms of biomass or cell-to-matrix ratios (Vanhommerig et al., 2014).

### Study design

First, we studied growth rates of each strain to understand if these differences might be due to differences in replication rates. Next, biofilm formation was assessed in a static biofilm model with a high-nutrient environment, followed by quantification after 24 h. Subsequently, these strains were allowed to form static biofilms for 24, 48, and 72 h with planktonic cultures generated in parallel. Samples for total RNA extraction were obtained at the given time points for both phenotypes and used for transcriptomic analyses. Genes found to be differentially expressed (at least 2-fold) between (i) the same strain’s biofilm and planktonic phenotypes at the same time point, or (ii) the same strain’s consecutive biofilm time points were studied further using metabolic pathway analysis. In parallel, *in silico* co-expression networks were constructed to observe inter- and intrastrain patterns.

Last, since it has been previously shown that presence of SCC*mec* influences MRSA biofilm formation (Pozzi et al., 2012), we investigated this further in the pandemic USA300 clone. Also, the arginine catabolic mobile element (ACME), which is unique to USA300, is shown to aid in skin colonization. Both SCC*mec* and ACME were excised to study their role in biofilm formation by USA300. Subsequently, the effect of single gene disruptions in ACME on biofilm formation was also investigated using *Bursa aurealis* transposon mutants.

### Bacterial growth conditions

All strains were grown either on 5% horse blood Columbia agar plates (Becton, Dickinson & Company, New York, NY, United States) or in brain heart infusion (BHI; Becton, Dickinson & Company, New York, NY, United States) broth supplemented with 0.1% D(+)-glucose monohydrate (Merck Millipore, Boston, MA, United States) as previously described (De Backer et al., 2018). *Bursa aurealis* transposon mutants in *S. aureus* USA300-JE2 were provided by the Network on Antimicrobial Resistance in *Staphylococcus Aureus* (NARSA) for distribution by BEI Resources (Nebraska Transposon Mutant Library (NTML),^1^ Supplementary Table 1; Fey et al., 2013).

### Growth rate determination and biofilm quantification

Growth rates of USA391, HEMRSA-15, and ST239-16 cultures were determined as previously described (De Backer et al., 2018). At each time point, average optical densities were calculated from quadruplicates. Additionally, biofilms of all three strains, as well as the NARSA transposon mutants, were formed on pre-sterilized 96-well flat-bottom polystyrene microtiter plates (CELLSTAR^®^, Greiner Bio-One, Kremsmünster, Austria) in triplicate to measure biofilm biomass using the crystal violet assay as described previously (De Backer et al., 2018).

### Biofilm and planktonic sample collection for transcriptomic analysis

All three strains, UAS391, HEMRSA-15, and ST239-16, stored at −80°C underwent two subsequent subcultures on blood agar (see section “Bacterial growth conditions” for media). A single colony was utilized to start an overnight broth and agar subculture from the same plate. The overnight broth culture was utilized to start and collect, in the exponential growth phase, 0.05 McF of each strain that was inoculated in a 136-mm polystyrene Petri dish (Greiner Bio-One, Austria), and grown under static conditions for 24, 48, or 72 h before rinsing (PBS, Thermo Fisher Scientific Inc., Boston, MA, United States) and scraping off adherent bacteria. Simultaneously, one colony from the blood agar subcultures was re-suspended in medium and incubated for 24, 48, or 72 h with shaking (250 rpm, 37°C) for each strain. The growth medium was refreshed every 24 h by spinning down the culture, removing the supernatant, and adding the fresh medium. After incubation, bacteria were collected by centrifugation at 4,600 × g for 20 min. Subsequently, approximately 0.200 g of both biofilm and planktonic bacterial suspensions were placed in RNAprotect™ Bacteria Reagent (Qiagen, Hilden, Germany) and incubated for 5 min at room temperature to stabilize the RNA. Bacteria were then harvested by centrifugation at 5,000 × g for 10 min at 4°C. Then two biological repeats were analyzed for each strain, phenotype, and time point.

### RNA isolation, library construction, and sequencing

Total RNA was purified from harvested bacteria using the Masterpure™ Complete DNA and RNA Purification kit (Epicentre^®^, Biotechnologies, Madison, WI, United States) according to the manufacturer’s protocol with modifications. Briefly, cells were mechanically disrupted using Lysing Matrix B 0.1 mm silica sphere bead beating (FastPrep^®^ 24 instrument, MP Biomedicals Inc., Irvine, CA, United States) before extraction. Each RNA sample was suspended in 30 μL of RNA storage solution, and the quality was determined using an Agilent 2100 Bioanalyzer (Agilent Technologies, Germany). Ribosomal RNA (rRNA) was depleted using a Ribo depletion kit (Thermo Fisher Scientific Inc., Netherlands), followed by preparation of a stranded TruSeq library and 1 × 75 bp sequencing using the NextSeq 500 system, v2 (Illumina Inc., San Diego, CA, United States).

### Transcriptome analysis

Raw reads were trimmed by Trimmomatic (v.0.39) (Bolger et al., 2014), and FastQC (v.0.11.8)^2^ was used for quality assessment. All processed data were normalized to the same sequencing depth (4,464,784 reads per sample) and then used for alignment by BBMap (v.38.47) against their reference genomes with default parameters to obtain transcripts per million (TPM) values (Bushnell, 2014). For UAS391 and HEMRSA-15, reference genomes (GenBank: CP007690.1 and CP007659.1, respectively) were obtained from the National Center for Biotechnology Information (NCBI). ST239-16 was whole-genome-sequenced in-house, trimmed as described previously, assembled by SPAdes (Bankevich et al., 2012), and annotated using Prokka (Seemann, 2014). DEGs (up- or downregulated) identified between (i) the biofilm and planktonic phenotype for each clone and (ii) in early (24 h), intermediate (48 h), and late (72 h) stages during biofilm formation were mapped to the Kyoto Encyclopedia of Genes and Genomes (KEGG) database of *S. aureus*. Expression levels of housekeeping and three randomly selected genes were validated using RT-PCR. Selected genes and used primers are listed in Supplementary Table 2.

### Construction and analysis of *in silico* co-expression networks

For each strain under biofilm or planktonic phenotypes, six samples representing the three time points (24, 48, and 72 h) were used to study temporal changes in gene (co-)expression by generating a network based on Spearman correlations with *p*_adj_ < 0.01 and |r| > 0.6. In total, six networks were generated for both the biofilm and planktonic phenotypes of the three strains. Networks were visualized by Gephi (v0.9.2) (Bastian et al., 2009), and their topological properties were calculated using the igraph package in R (v3.6) (Csárdi and Nepusz, 2006; R Core Team, 2020). In total, 1,000 Erdös–Réyni random networks, with equal numbers of nodes and edges as the corresponding observed network, were created using the igraph R package (Csárdi and Nepusz, 2006).

### Deletion of arginine catabolic mobile element and/or SCC*mec*

In UAS391, both ACME and SCC*mec* or single elements were cured by electroporation (20°C, 100 Ω, 25 μF, and 2.3 kV) of pSR2, a thermosensitive plasmid containing the *ccrAB2* gene complex and a tetracycline resistance marker, isolated from *Escherichia coli* SF8300 (ZymoPURE plasmid miniprep kit, Zymo Research, Irvine, CA, United States), using the method described by Katayama et al. (2000) and Diep et al. (2008). Here, the pSR2 plasmid was first transferred into the restriction-deficient intermediate *S. aureus* cloning host RN4220 to adapt the plasmid DNA to *S. aureus* modifications. RN4220 and UAS391 transformants were grown for three consecutive days at 30°C in BHI broth supplemented with tetracycline. Single colonies were picked and grown for 24 h in the BHI broth at 42°C to promote loss of pSR2. The deletion of ACME, SCC*mec*, or ACME and SCC*mec* was confirmed by PCR using X1-X5, X2-X3, and X1-X3 PCR primers (Diep et al., 2008). Additionally, transformants were screened for the presence of *arc* and *opp3* gene clusters by PCR-based assays, using the primer pairs AIPS.27 and AIPS.28 for *arcA*, and AIPS.45 and AIPS.46 for *opp3AB* (Diep et al., 2008). SCC*mec* typing was based on *ccr* recombinase and *mec* PCRs (Kondo et al., 2007).

### Statistical analysis

For RNASeq data, DESeq2 (v.1.14.1) was used to determine significantly differently expressed genes (DEGs) in each pairwise comparison based on TPM values of every gene (Love et al., 2014). Specifically, genes with *p* < 0.05 and fold change (FC) ≥ 2 were extracted, and their *p*-values were adjusted by using the false discovery rate (FDR) approach (p_adj_). Finally, genes with *p*_adj_ < 0.05 and FC ≥ 2 in each pairwise comparison were considered as DEGs. One-way ANOVA was conducted in R (v3.6) to evaluate the differences in biomass and growth rates at the three time points between the three tested strains. Transcriptomic profiles across both phenotypes and time points were compared using the permutational multivariate analysis of variance (PERMANOVA) using the Adonis function with 999 permutations in the vegan package^3^ in R.

## Results

### Gene expression of core genome pathways differs between methicillin-resistant *Staphylococcus aureus* strains/clones during early biofilm formation

In the static biofilm assay, HEMRSA-15 (OD492 = 0.856) and ST239-16 (OD492 = 1.366) showed higher biomass after 24 h of growth (+16% and +83%, respectively; *p* ≤ 0.03) than UAS391 (OD492 = 0.746, Supplementary Figure 1A). However, in the growth assay (OD620 measured after 24 h of growth at 37°C), the high biomass-forming HEMRSA-15 (0.149 min^–1^, doubling time: 4.65 min) and ST239-16 (0.160 min^–1^, doubling time: 4.31 min) showed a lower growth rate than UAS391 (0.164 min^–1^, doubling time: 4.21 min; *p* ≤ 0.046, Supplementary Figure 1B).

**FIGURE 1:**
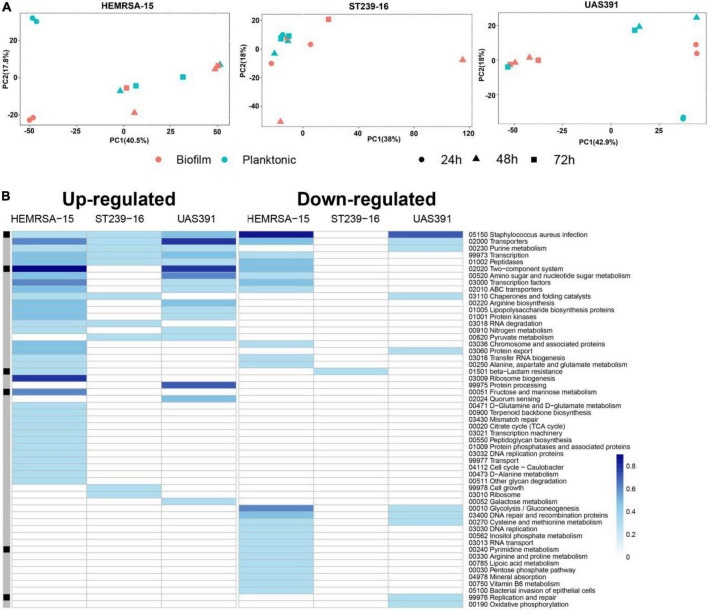
Gene expression analysis of 24-h biofilm and planktonic phenotypes in three representative MRSA strains: HEMRSA-15 (left), ST239-16 (middle), and UAS391 (right). **(A)** Principal component analysis shows global differences between biofilm (red) and planktonic (blue) phenotypes of all three strains. **(B)** Metabolic pathway signatures of biofilm compared to planktonic bacteria after 24 h of growth. Identified DEGs were mapped against the KEGG database to obtain KO terms and associated metabolic pathways. Color-coded cells represent the number of enriched genes [log_10_(1 + number of enriched genes)] in the respective KEGG pathway in accordance with the color scale. Black–gray marking indicates whether some enriched genes in the KEGG pathway are linked to the accessory genome (consisting of genes that are not present in all the three genomes, black) or in the core genome (gray). Two biological repeats were used for each phenotype in each strain across time points.

Principal component analysis in each of the three strains revealed substantial differences in transcriptional profiles between biofilm and planktonic phenotypes, as well as across the three time points (Figure 1A and Supplementary Tables 3, 4). After 24 h of growth, there were 120, 238, and 43 differentially expressed genes (DEGs) (p_adj_ ≤ 0.05 and FC ≥ 2) identified between the biofilm and planktonic phenotypes for UAS391, HEMRSA-15, and ST239-16, respectively (Supplementary Figure 2 and Supplementary Tables 5–7). This corresponded to 4.63, 9.29, and 1.54% of all CDSs, respectively. Among these genes, many hypothetical and phage-related genes were observed. As a control, the expression of five housekeeping genes (*gyrB*, *groE*, *glpF*, *gmk*, and *yqiL*) did not show significant differences between the biofilm and planktonic phenotypes in any of the three strains (*p*_adj_ > 0.05, Supplementary Table 8). Additionally, expression levels of randomly selected DEGs in the biofilm phenotype were confirmed by RT-PCR (Supplementary Figure 3).

All identified DEGs between the biofilm and planktonic phenotypes after 24 h of growth were mapped against the KEGG database with 56 (46.67%), 107 (44.96%), and 13 (30.23%) DEGs assigned KO terms for UAS391, HEMRSA-15, and ST239-16, respectively (Figure 1B). When comparing the gene expression of the top 10 mapped up and downregulated genes in biofilm with the planktonic phenotype in UAS391, HEMRSA-15, and ST239-16 at 24, 48, and 72 h, common genes were observed between UAS391 and HEMRSA-15 after 24 h of growth (Table 1). At 48 h, common genes were observed between UAS391 and ST239-16 but showing opposite expression patterns, while at 72 h, only one common gene between UAS391 and HEMRSA-15 was identified (Table 1). Genes involved in the KEGG pathways “*Staphylococcus aureus* infection,” “Transporters,” “Transcription,” and “Peptidases” were upregulated in all three biofilm phenotypes compared to their corresponding planktonic phenotypes. However, except these four KEGG pathways, transcriptomic signatures differed across the three strains. The up- and downregulated genes in UAS391 were involved in 17 and 10 KEGG pathways, respectively, in HEMRSA-15 in 34 and 24 KEGG pathways, respectively, and in ST239-16 in 10 and 1 KEGG pathways, respectively (Figure 1). UAS391 and HEMRSA-15 shared eight and five KEGG pathways with up- and downregulated genes, respectively, while HEMRSA-15 also showed 19 unique KEGG pathways, each with up- and downregulated genes. The total number of affected pathways in ST239-16 (*n* = 11) was remarkably lower than that in UAS391 (*n* = 27) and HEMRSA-15 (*n* = 58). Interestingly, the majority of the affected KEGG pathways were not linked to the accessory genome, indicating that expression of core genome pathways differed substantially in 24-h biofilms between the studied strains (Figure 1B).

**TABLE 1:** Top differentially expressed genes (up and down) with |log_2_(fold change)| ≥ 1 and adjusted *p*-value < 0.05 in biofilms compared to the planktonic phenotype after 24 h of growth of UAS391, HEMRSA-15, and ST239-16.

Gene annotation	UAS391	HEMRSA-15	ST239-16	KEGG pathway
**24 h**				
Chemotaxis protein	–2.36	–3.33		*Staphylococcus* aureus infection
Lactate dehydrogenase	–2.31	–2.45		Pyruvate metabolism
Hypothetical protein	–2.25			No classified
Complement inhibitor	–2.21			*Staphylococcus aureus* infection
Capsular biosynthesis protein	2.4			No classified
Carbamate kinase	2.41	3.95		Nitrogen metabolism
Lantibiotic epidermin	2.43			Two-component system
GTP pyrophosphokinase	2.45			Purine metabolism
Ornithine carbamoyltransferase	2.48	3.81		Arginine biosynthesis
Arginine deiminase	2.98	3.66		Arginine biosynthesis
Cysteine protease	3.98			Peptidases
Cold-shock protein	4.09	4.79		Transcription
Glutamyl endopeptidase	4.41			Quorum sensing
**48 h**				
General stress protein	–6.37			No classified
Glyceraldehyde-3-phosphate dehydrogenase	–5.69		3.33	Glycolysis/Gluconeogenesis
DNA-binding protein	–5.61		4.36	No classified
Cold-shock protein	–5.58		3.84	Transcription
Chaperone protein ClpB	–5.53		2.26	Longevity regulating pathway
Molecular chaperone DnaK	–5.46		3.09	RNA degradation
Acetoin reductase	–5.43		3.68	Butanoate metabolism
50S ribosomal protein L28	–5.42		5.51	Ribosome
Enolase	–5.35		3.64	Glycolysis/Gluconeogenesis
Quinol oxidase subunit 2	–5.24		3.37	Oxidative phosphorylation
Siderophore biosynthesis protein SbnD	2.05			Transporters
Transposase	2.15		–6.89	Replication and repair
dUTP pyrophosphatase	2.18			Pyrimidine metabolism
Pseudouridine-5’-phosphate glycosidase	2.24		–1.13	Pyrimidine metabolism
**72 h**				
General stress protein	–4.27	–1.26		No classified
50S ribosomal protein L28	–3.58			Ribosome
Lactate dehydrogenase	–3.42			Glycolysis/Gluconeogenesis
Glyceraldehyde-3-phosphate dehydrogenase	–3.38			Glycolysis/Gluconeogenesis
Preprotein translocase subunit SecY	–3.07			Protein export
DNA-binding protein	–3.05			No classified
Chaperone protein ClpB	–2.97			Longevity regulating pathway
50S ribosomal protein L22	–2.95			Ribosome
ATP:guanido phosphotransferase	–2.93			No classified
50S ribosomal protein L36	–2.93			Ribosome

Blue signifies upregulation, purple downregulation (|log_2_(fold change)| ≥ 1 and p_adj_ < 0.05), and white cells indicate the UAS391 genes were not identified as differentially expressed (DEGs) in HEMRSA-15 and/or ST239-16. Two biological repeats were used for each phenotype in each strain across time points.

Of note, in the “*Staphylococcus aureus* infection” pathway, the only DEG that was commonly upregulated in all three strains in 24-h biofilms was *clfA* (2.20-, UAS391; 2.07-, HEMRSA-15; and 2.66-fold, ST239-16; Table 2). The *clfA* gene encodes clumping factor A, an important virulence factor also involved in initial cell adherence. Other virulence genes such as those encoding the infection-related factors CHIPS (chemotaxis-inhibiting protein of *S. aureus*, *chs*), staphylokinase (*sak*), and a complement inhibitor SCIN family protein (*scn*) were downregulated in 24-h biofilms of UAS391 (−2.36-, −1.66-, and −2.21-fold, respectively) and HEMRSA-15 (−3.33-, −2.92-, and −3.20-fold, respectively) compared to planktonic bacteria (Table 2). Additionally, staphylococcal protein A (*lysM*) and gamma hemolysin subunit B (*hlgB*) showed downregulation in HEMRSA-15 (−2.11- and −2.33-fold, respectively, Table 2). In ST239-16 biofilms, *scn* and *lysM* were not differentially expressed compared to the planktonic phenotype (*p*_adj_ > 0.05), but virulence factors *chs*, *sak*, and *hlgB* were absent in the ST239-16 genome.

**TABLE 2:** Differentially expressed genes [log_2_(fold change)] in *S. aureus* infection-related pathways, ADI pathway, urea cycle, and high-affinity K^+^-specific transport system during biofilm growth compared to the planktonic phenotype of UAS391, HEMRSA-15, and ST239-16 MRSA.

	UAS391			HEMRSA-15			ST239-16		
			
Gene	24 h	48 h	72 h	24 h	48 h	72 h	24 h	48 h	72 h
***S. aureus* infection**									
* chs*	–2.36			–3.33					
* sak*	–1.66			–2.92					
* scn*	–2.11			–3.20					
* lysM*				–2.11					
* hlgB*				–2.33					
* clfA*	2.20			2.07			2.66		
**ADI-pathway**									
* arcA*	2.98	–2.80		3.66				2.38	
* arcB*	2.48	–1.29		3.81					
* arcC1*									
* arcC2_1*	2.41	–2.50		3.95	3.47			3.44	1.83
* arcC2_2*								3.63	
* arcD*	2.33	–1.61		3.67				3.10	
* arcR*	2.29	–1.87		3.68	3.31			3.49	
**Urea cycle**									
* argB*		1.56							
* argC*									
* argF*									
* argG*									
* argH*				1.38					
* argJ*		1.52							
* rocD2_1*		1.41							
* rocD2_2*									
* rocF*									
* ureA*	2.33	–1.73							
* ureB*	2.18								
* ureC*	1.99	–1.27							
* ureD*	2.32	–1.54							
* ureE*	2.37	–1.20							
* ureF*	2.35	–1.94							
* ureG*	2.01	–1.65							
**High-affinity K^+^-specific transport system**									
* kdpA*	2.25			2.61					
* kdpB*	2.23			3.17					
* kdpC*	2.29	–1.64		3.01	–1.52			1.45	
* kdpD*				1.08					
* kdpE*				1.94					

Blue signifies upregulation, purple downregulation (|log_2_(fold change)| ≥ 1 and p_adj_ < 0.05), white cells indicate no DEG was detected, and gray cells indicate the gene was not present. Two biological repeats were used for each phenotype in each strain across time points.

### The arginine deiminase pathway is upregulated during biofilm formation by methicillin-resistant *Staphylococcus aureus*

Since we previously showed the influence of the arginine-synthesizing, urea, and tricarboxylic acid (TCA) cycles on USA300 biofilm formation (De Backer et al., 2018), arginine-related pathways were further investigated here. Under anoxic conditions, found in biofilms, arginine can provide energy in the form of ATP through catabolism with the formation of ammonia through the arginine deiminase (ADI) pathway, or be utilized as a substrate in the urea cycle with additional ammonia generation (Cunin et al., 1986). In 24-h biofilms of UAS391 and HEMRSA-15, the ADI pathway (*arcA*, *arcB*, *arcC2_1*, *arcD*, and *arcR*) was upregulated at least 2- and 3-fold, respectively, compared to the planktonic phenotype (Table 2). Among these, *arcA* and *arcC2_1* were most highly upregulated (2.98- and 3.95-fold, respectively) in UAS391 and HEMRSA-15 24-h biofilms. In line with these results, transposon inactivation of *arcA* in USA300-JE2 proved to have a significant impact on produced biofilm, compared to the wild type (OD492 = 0.372, 57%; *p* ≤ 0.001, Supplementary Figure 4). Moreover, *arcC_2* inactivation decreased the biofilm production to an equal level, as observed in the *arcA*:Tn mutant (OD492 = 0.373, 57%; *p* ≤ 0.001, Supplementary Figure 4). Contrasting the UAS391 and HEMRSA-15 biofilms, in ST239-16 biofilm, the upregulation of the ADI pathway was only observed after 48 h of biofilm growth with all elements, except *arcB*, showing a minimum of 2-fold upregulation compared to planktonic cells (Table 2).

Arginine can be synthesized in the arginine biosynthesis pathway (*argBCJ*) coupled with the urea cycle (*argFGH*, *rocF*) (Cunin et al., 1986). Expression levels were stable in 24-h biofilm phenotypes of all three strains with no DEGs detected in both pathways when comparing to respective planktonic phenotypes (Table 2). On the other hand, in 24-h biofilms, differential expression was observed for genes related to urease (*ureABCDEFG*), which catabolizes urea formed by arginase (*rocF*) in the urea cycle. In UAS391 24-h biofilms, all urease-related genes showed FCs ranging from 1.99 to 2.37 (Table 2). Another arginine-synthesizing pathway, the pyrimidine nucleotide biosynthesis pathway (*pyrRPBC*, *carAB*, and *pyrFE*), did not show significant differential expression in 24-h biofilms of the three strains (*p* > 0.05).

In addition to arginine catabolism-mediated ammonia production, the high-affinity K^+^-specific transport system (*kdpABC* and *kdpDE*) also contributes to pH homeostasis through the exchange of K^+^ for H^+^ (Cotter and Hill, 2003). Also here, 24-h biofilms showed differential expression when compared to its planktonic counterpart: in UAS391, *kdpABC* was upregulated (2.25-, 2.23-, and 2.29-fold, respectively), and in HEMRSA-15, both *kdpABC* and *kdpDE* were upregulated (2.61-, 3.17-, 3.01-, 1.08-, and 1.94-fold, respectively). By contrast, in the ST239-16 24 h biofilm, this was not observed (Table 2).

### Maturing biofilms show distinct temporal changes in gene expression patterns

**FIGURE 2:**
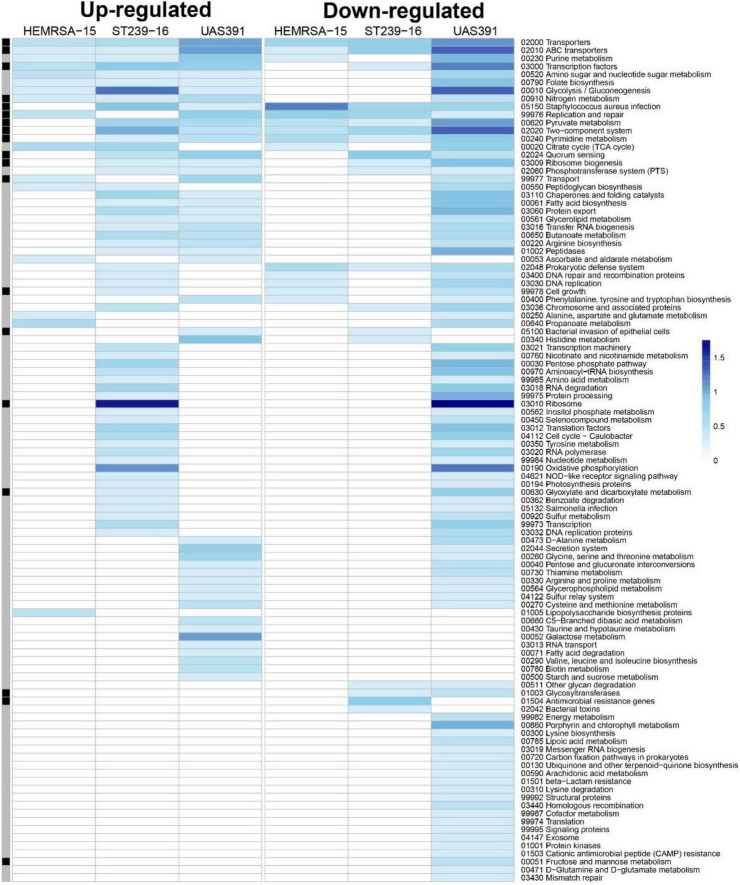
Gene expression analysis of 48-h biofilm and planktonic phenotypes in three representative MRSA strains: HEMRSA-15 (left), ST239-16 (middle), and UAS391 (right). Metabolic pathway signatures of biofilms compared to planktonic bacteria after 48 h of growth. Identified DEGs were mapped against the KEGG database to obtain KO terms and associated metabolic pathways. Color-coded cells represent the number of enriched genes [log_10_(1 + number of enriched genes)] in the respective KEGG pathway in accordance with the color scale. Black–gray marking indicates whether some enriched genes in the KEGG pathway are linked to the accessory genome (black) or not (gray). Two biological repeats were used for each phenotype in each strain across time points.

As with 24-h biofilms, gene expression patterns in 48- and 72-h biofilms showed substantial differences from their planktonic counterparts, as shown by PCA (Figure 1A). Of all identified DEGs, KEGG mapping could be performed for 595 (65.7%), 70 (25.4%), and 250 (49.3%) of the identified DEGs for the 48-h time point for UAS391, HEMRSA-15, and ST239-16, respectively (Figure 2). After 72 h of growth, the number of mapped DEGs were limited to 72 (82.8%), 2 (20%), and 11 (40.7%) for the three strains, respectively (Supplementary Figure 5). As with the 24-h time point, transcriptomic signatures differed remarkably between strains; especially, UAS391 showed a significant amount of affected KEGG pathways, which were unaffected in the two other strains (Figure 2). In contrast to the 24-h time point, more affected pathways were linked to the accessory genome. These pathways were also affected in two or all three strains, indicating this change in gene expression was common across all three strains. Also noticeable were a number of core genome-linked KEGG pathways, which were downregulated in UAS391 but upregulated in ST239-16, again indicating differences in gene expression patterns between the strains (Figure 2).

Comparing temporal biofilm time points within strains, differential expression analysis showed high numbers of DEGs: 48-h biofilm to 24-h biofilm [653 (UAS391), 563 (HEMRSA-15), and 422 (ST239-16)] and 72-h biofilm to 48-h biofilm [3 (UAS391), 198 (HEMRSA-15), and 406 (ST239-16), Supplementary Figure 6]. Housekeeping genes did not show significant differences between time points, and RT-PCR confirmed expression levels of random DEGs (Supplementary Table 9 and Supplementary Figure 3). Furthermore, null model testing showed that the global gene expression patterns were non-stochastic and therefore could not be attributed to random fluctuations at the single-gene level (Supplementary Table 10).

However, as with expression patterns in 24- and 48-h biofilms compared to the planktonic phenotype, ST239-16 showed striking differences during biofilm maturation compared to the other strains (Supplementary Figure 6). In the 24- to 48-h biofilm timeframe, expression levels of several metabolism pathways showing downregulation in UAS391 and HEMRSA-15, such as glycolysis/gluconeogenesis and oxidative phosphorylation, showed upregulation in ST239-16 (Supplementary Figure 6). Also, genes encoding ribosomal proteins and the biogenesis of ribosomes showed this trend with 65 and 56 downregulated genes between 24- and 48-h biofilms for UAS391 and HEMRSA-15, respectively, but 41 upregulated genes in ST239-16 (Supplementary Figure 6).

The ADI pathway, showing significant upregulation in 24-h biofilms of UAS391, ST239-16, and HEMRSA-15 compared to planktonic cells, did not show differential expression in biofilms of 48 h and 72 h for both strains (Table 2). However, when comparing 48-h UAS391 biofilm to 48-h planktonic cells, all components were downregulated in the biofilm phenotype with FC ranging from −1.29 to −2.8-fold (Table 2). In HEMRSA-15, this pattern was not observed. Similar results were observed for urease-related genes, upregulated in 24-h UAS391 biofilm, with most components downregulated comparing 48-h UAS391 biofilm to 24-h biofilm (FC: −1.49 to −2.08-fold) and 48-h UAS391 planktonic (FC: −1.2 to −1.94-fold, Table 3), suggesting a distinct role in early UAS391 biofilm formation.

**TABLE 3:** Differentially expressed genes [log_2_(fold change)] of the urea cycle, pyrimidine biosynthesis, high-affinity K^+^-specific transport system, and ACME-associated genes (only in UAS391) in a biofilm of UAS391, HEMRSA-15, and ST239-16 MRSA.

	UAS391		HEMRSA-15		ST239-16	
			
Gene	48 h	72 h	48 h	72 h	48 h	72 h
**Urea cycle**						
* ureA*	–1.64					
* ureB*	–1.49					
* ureC*						
* ureD*	–2.08					
* ureE*	–1.57					
* ureF*	–1.99					
* ureG*	–1.92					
**Pyrimidine biosynthesis**						
* carA*	–2.01					
* carB*	–1.90					
* pyrB*	–1.71					
* pyrC*	–1.95					
* pyrE*	–1.76		–1.01			
* pyrF*	–2.01		–1.07		1.08	
* pyrP*						
* pyrR*	–3.61				1.38	
**High-affinity K^+^-specific transport system**						
* kdpA*	–2.09		–2.03			
* kdpB*	–1.91		–1.78			
* kdpC*	–2.66		–2.45		1.55	–1.35
* kdpD*						
* kdpE*	–1.61		–1.99			
**ACME element**						
* speG*						
* arcA*	1.66					
* arcB*	2.43					
* arcC*	3.01					
* arcD*	1.69					
* argR*						
* opp-3A*						
* opp-3B*						
* opp-3C*						
* opp-3D*						
* opp-3E*						

Expression levels indicate fold changes in comparison to the previous time point. Blue signifies upregulation, purple downregulation (|log_2_(fold change)| ≥ 1 and p_adj_ < 0.05), and white cells indicate no DEG was detected. Gray cells indicate the gene was not present. Two biological repeats were used for each phenotype in each strain across time points.

Interestingly, the pyrimidine nucleotide biosynthesis pathway, unaffected in 24-h biofilms, showed significant downregulation of all components (except *pyrP*) in 48-h UAS391 biofilms, compared to the 24-h time point, with FC ranging from −1.71 to −3.61 (Table 3). By contrast, HEMRSA-15 and ST239-16 48-h biofilms only showed differential expression of two components: *pyrFE* (−1.07- and −1.01-fold, respectively) and *pyrF* and *pyrR* (1.08- and 1.38-fold, respectively, Table 3). On the other hand, the high-affinity K^+^-specific transport system, upregulated in UAS391 and HEMRSA-15 24-h biofilms compared to planktonic cells, showed subsequent downregulation in 48-h biofilms (Table 2). *KpdABC* and *kpdE* showed negative FCs when comparing 48-h with 24-h biofilms for both strains. In the 72-h biofilms, none of the genes was identified as DEGs (Table 3).

### Gene co-expression network analysis

Topological properties in the observed networks significantly differed from those in the corresponding random networks (*p* < 0.05, one-sample *t*-test, Supplementary Table 10), indicating that the observed patterns of gene co-expression along the time points (24, 48, and 72 h) in each strain were not stochastic, in line with the null model results that the observed transcriptional profiles significantly differed (*p* < 0.01) from the expected profiles (Supplementary Table 11). Positive connections dominated in all networks, indicating genes showed similar expression patterns along the time points. Gene co-expression patterns were more similar between UAS391 and HEMRSA-15, with higher network connectivity (indicated by average degree, and numbers of nodes and edges) than ST239-16 networks. In UAS391 and HEMRSA-15, there was lower network connectivity in biofilms than in planktonic networks, while an opposite pattern was observed in ST239-16 networks. Inside the modules of the UAS391 and HEMRSA-15 networks, some specific pathways such as ribosomal protein synthesis/activity, sugar metabolisms, and/or even oxidative phosphorylation dominated. However, no domination of specific pathways in the ST239-16 module was observed (Supplementary Figure 7).

### Carriage of an additional arginine deiminase cluster on the arginine catabolic mobile element

To observe the impact of the ACME, which is uniquely carried by USA300, on biofilm formation, we excised the element, which resulted in a small deficit in biofilm formation capacity (OD492 = 0.619, 83%, *p* = 0.13), as compared to the wild type (Supplementary Figure 8A). Knocking out each gene on the ACME revealed a large biofilm decrease when *opp3D* was disrupted in USA300-JE2 (OD492 = 0.372; 57%; *p* ≤ 0.05, Supplementary Figure 8B), while other transposon disruptions only resulted in small or no biofilm defects. Since our results showed a significant upregulation of the ADI pathway in 24-h biofilms, it was also of interest that ACME harbors an additional ADI gene cluster. We observed upregulation of *arcA*, *arcB*, *arcC*, and *arcD* in UAS391 biofilms. Here, *arcABCD* was on average 2-fold upregulated in 48-h biofilms, compared to the 24-h time point (Table 3).

Arginine catabolic mobile element is closely associated with methicillin resistance encoding SCC*mec*, where transcriptomic data showed no differential expression of SCC*mec* genes between biofilms and planktonic phenotypes in all three strains (*p* > 0.05). However, lower expression of *mecA* and *mecR1* components was observed in later biofilm time points (48 and 72 h) compared to 24 h in UAS391 and HEMRSA-15. The deletion of SCC*mec*, as with ACME, resulted in a small biomass decrease of 18% (OD492 = 0.429, 82%; *p* = 0.22, Supplementary Figure 8A). On the other hand, combined deletion of both elements resulted in a much larger biofilm defect (OD492 = 0.614, 58%; *p* < 0.001).

## Discussion

Understanding the ability of pandemic MRSA clones causing human infections to form biofilms and the mechanisms underlying biofilm formation is the first step toward a possible solution for biofilm-related infections. However, our temporal biofilm transcriptome analysis of three major MRSA clones causing infections, USA300, HEMRSA-15, and ST239-16, showed remarkable divergence in gene expression patterns and in metabolic pathways involved in biofilm formation. Although this study only tested a single strain per clonal type, the fact that temporal changes in gene expression during biofilm formation were primarily observed in core genome-encoded pathways supports our hypothesis that these data could be broadly representative of the three major clones studied here.

Early (24-h) biofilms primarily showed pathways encoded in the core genome to be affected in all three clones, and these included KEGG pathways “*Staphylococcus aureus* infection,” “Transporters,” “Transcription,” and “Peptidases.” However, the only gene that was commonly upregulated across all three biofilms was *clfA*, which encodes clumping factor A (ClfA), a cell wall-anchored protein and a virulence factor that promotes bacterial adhesion to the blood plasma protein fibrinogen even under high shear stress and facilitates colonization by *S. aureus* of blood protein-coated biomaterials (Herman-Bausier et al., 2018). Interestingly, a high-affinity anti-ClfA monoclonal antibody has already been explored as a potential target for prosthetic joint and surgical site infections caused by *S. aureus* (Mao et al., 2021). Prior studies that have performed global transcriptomic analysis of *S. aureus* biofilm formation, including a 24-h time point, have shown differing results (Atshan et al., 2013; Tomlinson et al., 2021). For instance, Atshan *et al.* utilized qPCR on 12 gene targets at 6, 12, 24, and 48 h and compared the latter three time points to the 6-h biofilms on the premise that this calibrator sample did not show any cell-associated fibrous material (Atshan et al., 2013). ClfA is known to be expressed early in biofilms, and Atshan et al. (2013) did not find any significant difference between 6- and 12-h biofilms for *clfA*, although there was a significant decrease in expression at 24 and 48 h compared to the 6-h samples. We, on the other hand, found *clfA* upregulation in 24-h biofilms compared to planktonic cultures of the same strain grown head-to-head for the same length of time, indicating that up regulation of this gene is a marker of a lifestyle switch in MRSA. The study by Tomlinson et al. (2021) is closer to ours in terms of study design, although their conclusion of the role of *clfA* in late-stage maturation and strengthening of biofilms was based on the fact that they studied 5-, 10-, and 24-h biofilms, and the lattermost time point was considered as the late-stage biofilm in contrast to our study, where a 24-h biofilm was defined as an early stage biofilm.

Given the strong relationship between biofilm and *S. aureus* infections, the expression of related virulence factors was compared. Staphylokinase has been shown to disrupt biofilm formation in both *S. aureus* and polymicrobial biofilms (Kwiecinski et al., 2016; Liu et al., 2019) and to induce detachment (Liu et al., 2019). In accordance, our data showed a downregulation of the staphylokinase-encoding *sak* in 24-h biofilms of UAS391 and HEMRSA-15. As expected, several virulence factors were absent from ST239-16. HA-MRSA is known to harbor a lower number of virulence factors and also exhibit lower expression of core virulence factors than CA-MRSA strains (Otto, 2013; Wang et al., 2016).

In biofilms grown for 48 and 72 h, we found a change in the expression of genes mainly associated with central metabolic and fermentation pathways, suggesting a shift to anaerobic conditions (Fuchs et al., 2007). However, while the phenomenon of down regulation of central metabolic pathways (glycolysis/gluconeogenesis, fatty acid biosynthesis, oxidative phosphorylation, and others) was already evident in UAS391 and HEMRSA-15 in 48-h compared to 24-h biofilms, ST239-16 showed the same at 72 h. In fact, at 48 h, ST239-16 biofilms showed up regulation of ribosomal proteins indicative of actively dividing cells with the delayed switch to the classical biofilm phenotype only at 72 h.

Under anaerobic conditions, some bacteria can generate ATP as an energy source through catabolism of arginine *via* the ADI pathway (Cunin et al., 1986). Collectively, the *arc* genes convert arginine into ornithine, ammonia, and carbon dioxide, yielding 1 mol of ATP per mol of arginine. Indeed, our data showed strong up regulation of the *arcABDCR* operon in 24-h biofilms of UAS391 and HEMRSA-15, and in 48-h biofilms of ST239-16, and a critical role of arginine deiminase ArcA, where inactivation led to a distinct decrease in biofilm formation by USA300-JE2 at 24 h. The contribution of ArcA and the ADI pathway has previously been reported in *Staphylococcus epidermidis* (Lindgren et al., 2014) and, recently, *Streptococcus pyogenes* (Freiberg et al., 2020). Moreover, we also found a second ADI pathway-encoded gene, carbamate kinase (ArcC), which transfers phosphate from carbamoyl phosphate to ADP, to be a key regulator of biofilm formation. As observed with *arcA*, Tn insertion in *arcC* also led to a similar 43% decrease in biofilm formation by USA300-JE2 at 24 h. Carbamoyl phosphate synthetase, encoded by *carAB*, is also utilized in the pyrimidine biosynthesis pathway, which is encoded by *pyrRPBC* and *pyrFE*. These genes showed significant downregulation in 48-h UAS391 biofilm, compared to the 24-h time point, suggesting a role of carbamoyl-phosphate in several pathways during fermentative growth. Interestingly, the other two MRSA did not show this differential expression in their biofilm, highlighting differences in their metabolic activity. Our data indicate a critical role of arginine catabolism pathways in early biofilm formation.

In addition to ATP generation, the ADI pathway can also function as an ammonia-generating pathway, by the deimination of arginine, which is utilized by oral biofilm-forming bacteria such as *Streptococcus salivarius* for pH homeostasis (Li et al., 2000). However, Zhu et al. (2007) demonstrated that ammonia generated by the ADI pathway is insufficient to counteract the drop in pH due to the accumulation of organic acids. A second ammonia-producing mechanism counteracting acidification is urease (*ureABCDEFG*), which catabolizes urea produced in the urea cycle to ammonia and CO_2_. As observed in the pyrimidine biosynthesis pathway, distinct differential expression was observed in UAS391, but not in HEMRSA-15 and ST239-16. UAS391 showed high upregulation of the urease genes in 24-h biofilms, countered by a subsequent downregulation in biofilms, indicating its importance in early biofilm formation of this clone. Bacterial ureases have been described as a potential key factor in the persistence of certain pathogens, with the release of ammonia being the major cause of host tissue damage (Burne and Chen, 2000). Urease activity is known to protect bacteria in acidic environments (Mobley et al., 1995), which was seen in *S. aureus* when put under acid shock (Bore et al., 2007). Last, the coupled release of CO_2_ also influences bacterial physiology, and intra- and extracellular pH homeostasis, while buffering the biofilm itself to protect bacteria encapsulated in it (Burne and Chen, 2000).

Our data also showed upregulation of the high-affinity K^+^-specific transport system (*kdpABCDE*), apparently contributing to pH homeostasis by cation transport (Cotter and Hill, 2003). This operon was upregulated in HEMRSA-15 (complete operon) and UAS300 (*kdpABC*) 24-h biofilms. Similar to the urease pathway, this early trend was not observed in biofilms of both strains, while ST239-16 only showed upregulation of *kdpC* in 48-h biofilms. The differences in gene expression between biofilm and planktonic cells are determined by distinct phenotypes but also might be partly attributed to differences in micro-environments (e.g., nutrient and waste concentrations) between biofilm and planktonic cell cultures.

Last, we showed that a deletion of both ACME and SCC*mec* mobile elements led to a significant decrease in biomass formation compared to deletion mutants of single elements, suggesting a link between the two elements which are harbored as a composite element in the USA300 clone. Pozzi et al. (2012) reported biofilm defects when excising SCC*mec* from clinical MRSA isolates but observed heterogeneity among isolates with different genetic backgrounds (clonal complex and SCC*mec* type). Expression data of the SCC*mec* genes did not show significant differences between biofilm and planktonic phenotypes. Single transposon interruptions of ACME-related genes showed no influence of the ADI genes. However, this could be explained by the fact that this is an additional copy of the ADI genes in strains belonging to USA300, and loss could be compensated by the putative ADI -cluster. On the other hand, disruption of *opp3D*, an ABC transporter ATP-binding protein harbored on the ACME, had a significant negative impact on biofilm formation by USA300-JE2.

In conclusion, we show here striking differences in gene expression during biofilm formation by representative strains of three globally important MRSA clones, USA300, HEMRSA-15, and the Hungarian/Brazilian ST239 clone. Notwithstanding the fact that ST239-16 has a larger accessory genome (12.18%) than UAS391 (5.12%) or HEMRSA-15 (7.75%), the majority of affected pathways in early, 24-h biofilms involved the core genome. The *clfA* gene was the only significantly upregulated gene across all three strains in 24-h biofilms and exemplifies an important target to disrupt early biofilms. The fact that neutralizing human monoclonal antibodies against *ClfA* were found to inhibit biofilm formation *in vitro* and the hematogenous implant-related infection *in vivo* (Wang et al., 2017) strengthens the validity of our *in vitro* results. However, given the wide variations among MRSA lineages, further research into genomic and associated phenotypic characteristics of other MRSA lineages is required to enable generalization of our results.

## Data availability statement

The datasets presented in this study can be found in online repositories. The names of the repository/repositories and accession number(s) can be found below: https://www.ncbi.nlm.nih.gov/, PRJNA769537.

## Author contributions

HD, J-PH, HG, and SM-K: conceptualization. JV, QL, BX, and SDB: investigation. JV and QL: visualization. JV, QL, SDB, and SM-K: writing—original draft preparation. JV, QL, BX, MB, HD, J-PH, SK-S, HG, and SM-K: writing—review and editing. All authors read, gave input, and approved the final manuscript.

## Footnotes

1
https://www.beiresources.org

2
https://www.bioinformatics.babraham.ac.uk/projects/fastqc/

3
https://github.com/vegandevs/vegan
