# Supplementary material for: The dynamic transcriptome during maturation of biofilms formed by methicillin-resistant Staphylococcus aureus
Source: Front Microbiol. 2022 Jul 28;13:882346. doi: 10.3389/fmicb.2022.882346 (PMC9366926; doi:10.3389/fmicb.2022.882346)
Supplement: Supplementary file 1 [file Data_Sheet_1.docx]

Supplementary Material

# Supplementary Figures and Tables

## Supplementary Figures


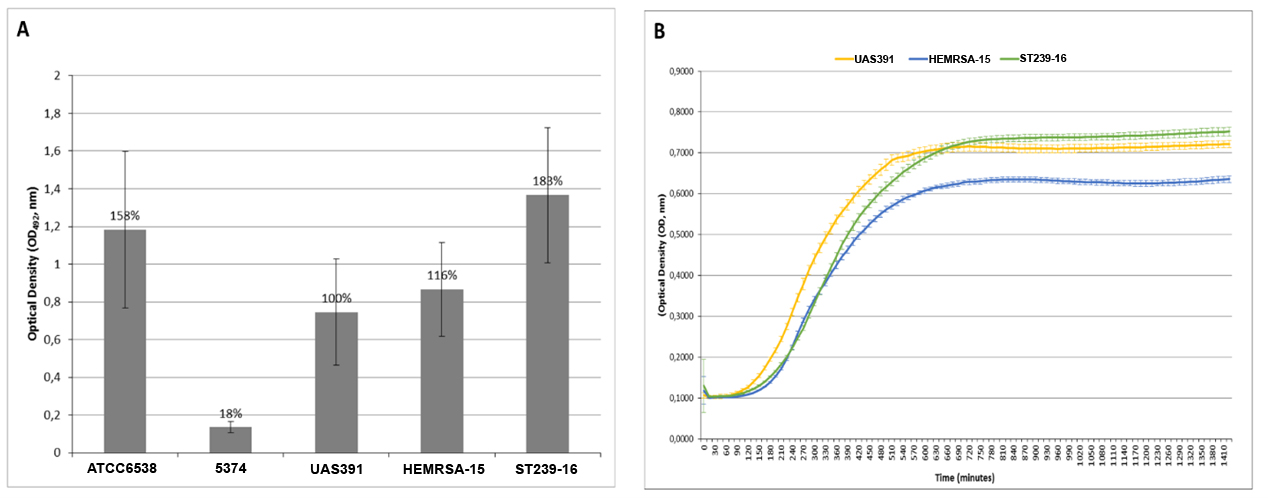


**Supplementary Figure 1.** Phenotypic characterization of MRSA strains UAS391, HEMRSA-15, and ST239-16. **(A)** Formation of biofilms by UAS391, HEMRSA-15, and ST239-16 under static conditions. Error bars represent 95% confidence intervals. ATCC6538 and 5374 were included as positive and negative controls, respectively. **(B)** Background absorption-corrected 24h growth curves for UAS391, HEMRSA-15, and ST239-16. Error bars in corresponding color represent the 95% confidence interval per strain.


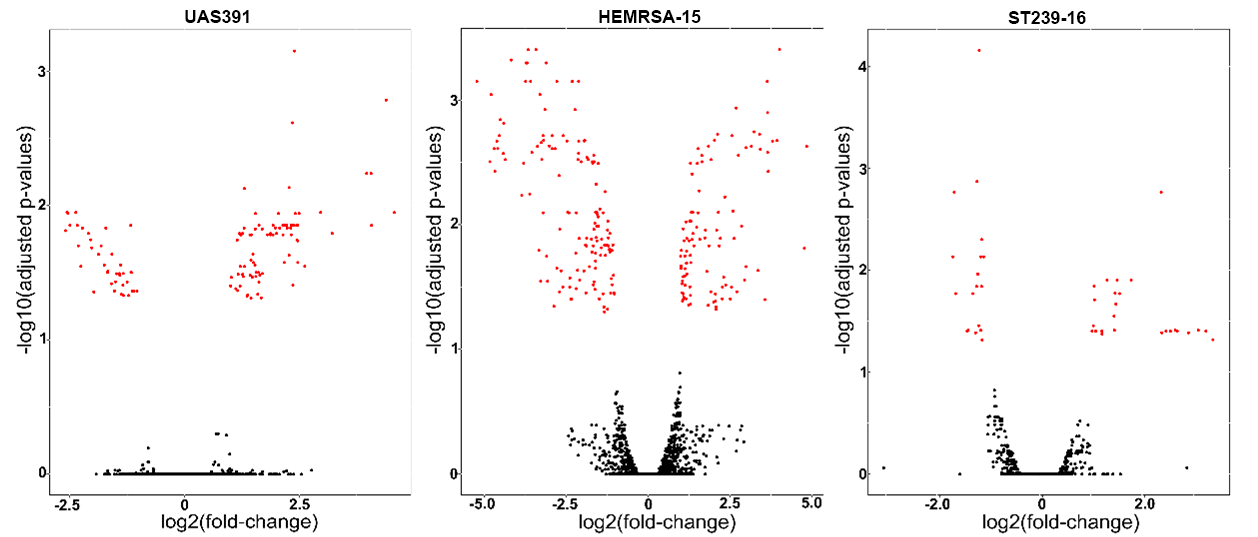


**Supplementary Figure 2.** Volcano plots showing differential gene expression patterns in 24h biofilm compared to 24h planktonic bacteria for UAS391 (left), HEMRSA-15 (middle), and ST239-16 (right). The log_2_(FC) is plotted on the X-axis and the negative log_10_(p_adj_) is plotted on the Y-axis. Black dots indicate no differential gene expression with the absolute value of log_2_(FC) < 1 or p_adj_ ≥ 0.05. Red dots indicate differentially expressed genes with the absolute value of log_2_(FC) ≥ 1 and p_adj_ < 0.05).


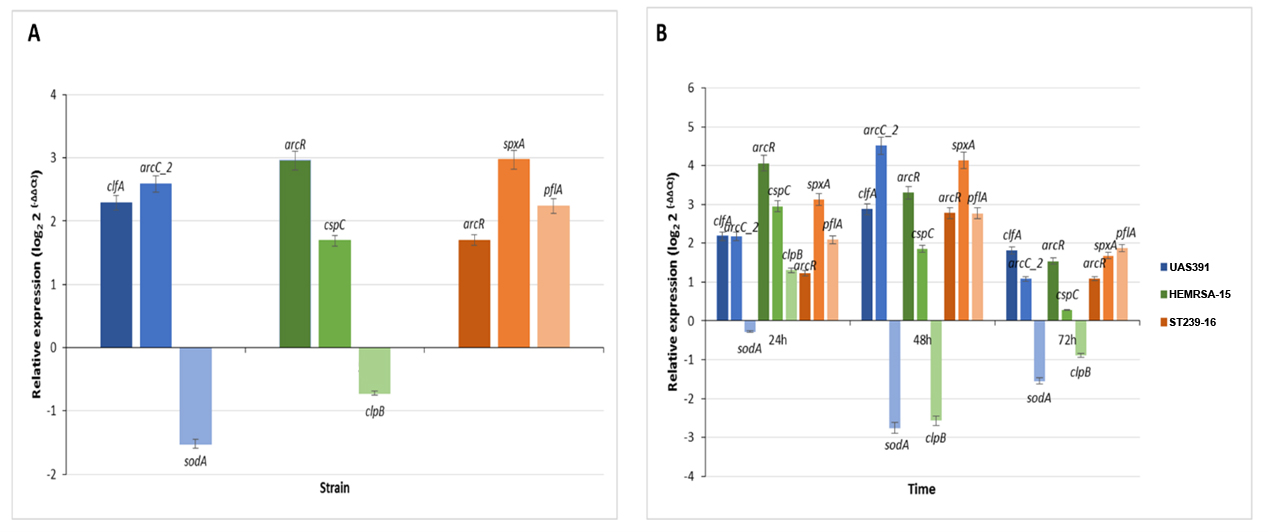


**Supplementary Figure 3.** Quantification of relative gene expression in UAS391, HEMRSA-15, and ST239-16. *clfA* (clumping factor A), *arcC_2* and *sodA* (superoxide dismutase (Mn/fe family)), *arcR*, *cspC* (cold shock protein), *clpB* (chaperone clpB), *arcR*, *spxA* (regulatory protein Spx), and *pflA* (pyruvate formate-lyase activating enzyme) relative gene expression in UAS391, HEMRSA-15 and ST239-16, respectively. **(A)** Average relative gene expression in planktonic cells of UAS391, HEMRSA-15, and ST239-16 was taken as baseline 0. **(B)** Relative gene expression in 24h, 48h, and 72h planktonic cells UAS391, EMRSA-15, and ST239-16 were taken as baseline 0.


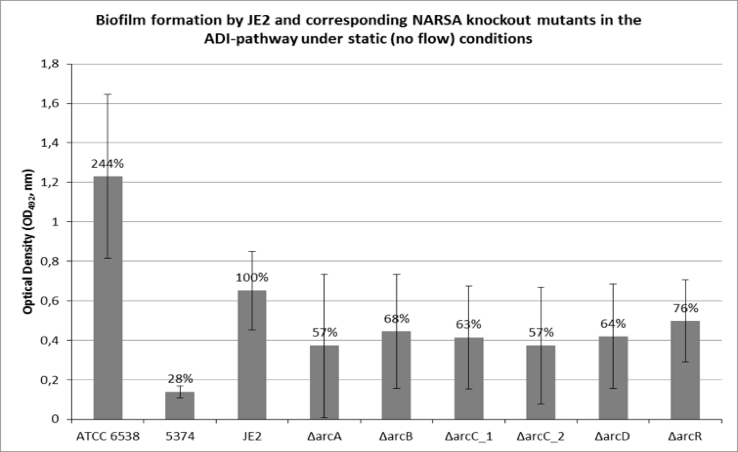


**Supplementary Figure 4.** Formation of biofilms by USA300-JE2 and corresponding transposon knock-out mutants in ADI-pathway encoding genes under static conditions. Error bars represent 95% confidence intervals. ATCC6538 and 5374 were included as positive and negative controls, respectively. *arcA*: arginine deiminase, *arcB*: ornithine carbamoyltransferase, *arcC_1* & *arcC_2*: carbamate kinase, *arcD*: arginine/ornithine antiporter, *arcR*: positive transcriptional regulator.


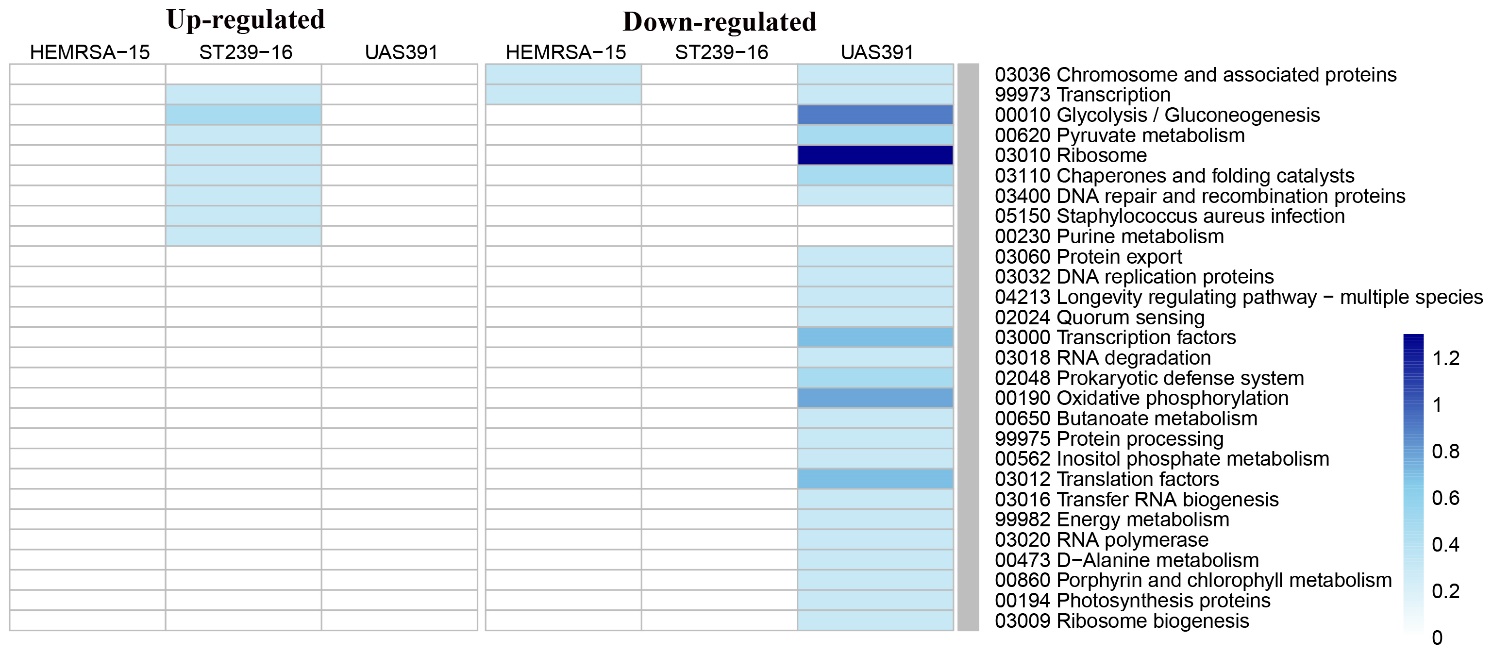


**Supplementary Figure 5**. Gene expression analysis of 72h biofilm and planktonic phenotype in HEMRSA-15 (left), ]ST239-16 (middle), and UAS391 (right). Metabolic pathway signatures of biofilm compared to planktonic after 72h of growth. Identified DEGs were mapped against KEGG database to obtain KO terms and associated metabolic pathways. Color coded cells represent the number of enriched genes (log_10_ (1+number of enriched genes)) in the respective KEGG pathway in accordance to the color scale. Black-grey marking indicates whether some enriched genes in the KEGG pathway are linked to the accessory genome (black) or not (grey).


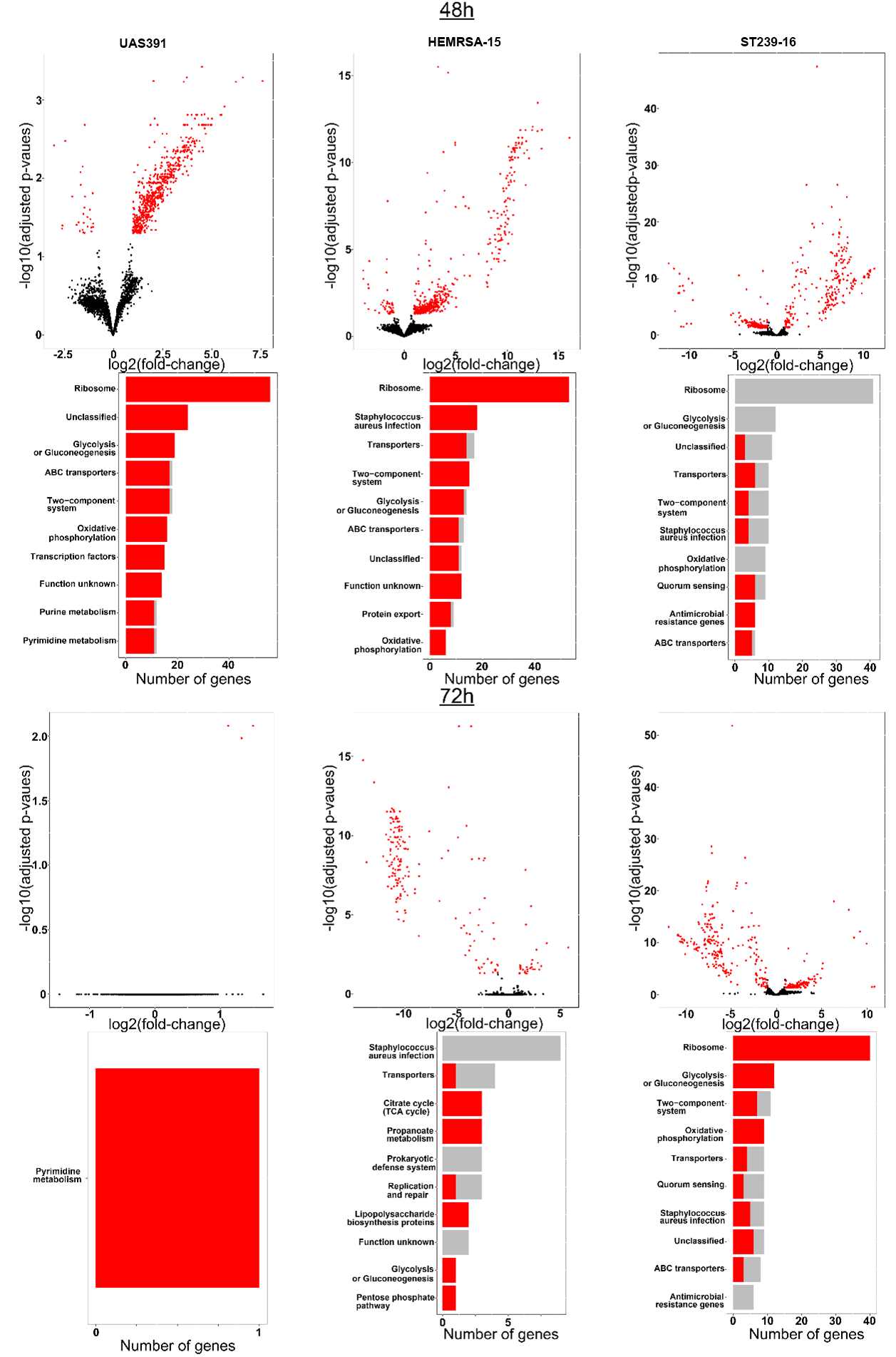


**Supplementary Figure 6**. Gene expression analysis of ageing biofilms UAS391 (left), HEMRSA-15 (middle), and ST239-16 (right). Volcano plots showing differential gene expression patterns and KEGG pathway analysis of identified DEGs showing the top 10 affected KEGG pathways in 48h biofilms compared to 24h biofilms (Top) and 72h biofilms compared to 48h biofilms (Bottom) for all three clones. For the volcano plots the log_2_(FC) is plotted on the X-axis and the negative log_10_(p_adj_) is plotted on the Y-axis. Black dots indicate no differential gene expression with the absolute value of log_2_(FC) < 1 or p_adj_ ≥ 0.05. Red dots indicate differentially expressed genes with the absolute value of log_2_(FC) ≥ 1 and p_adj_ < 0.05. For the KEGG pathway analysis, the Y-axis represents affected KEGG pathways and the X-axis the number of genes identified as DEG. Red signifies down-regulation in the later time point, grey upregulation.


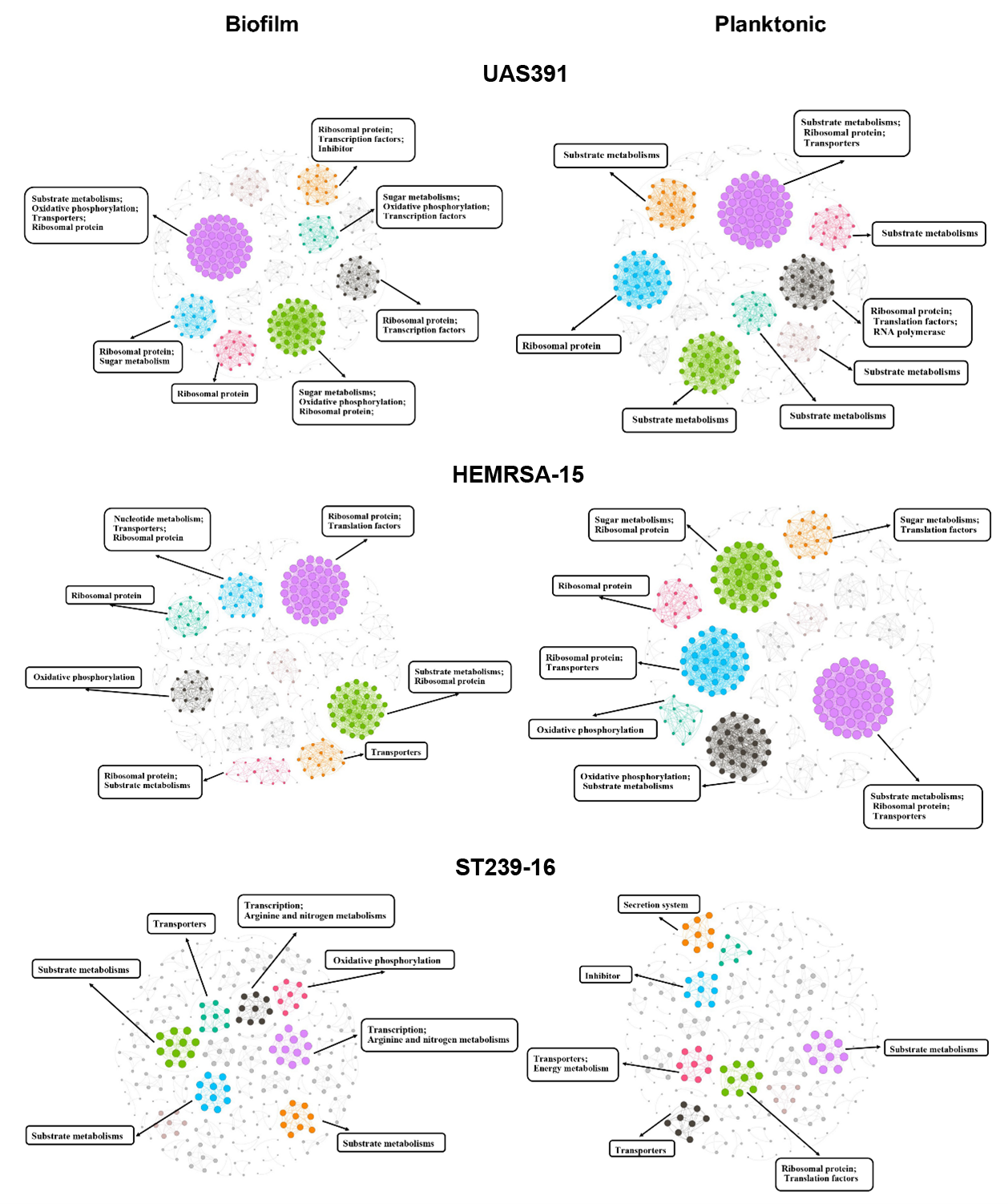


**Supplementary Figure 7.** Networks of gene co-expression during biofilm and planktonic cultures of pandemic UAS391 (Top), HEMRSA-15 (Middle), and ST239-16 (Bottom) MRSA clones. Network modules are colored and marked by pathways mainly included in modules. Network nodes are sized by degree. Only modules, including nodes >7 were marked. “Substrate metabolisms” means metabolisms of various substrates, but none of the substrates are dominated in the module.


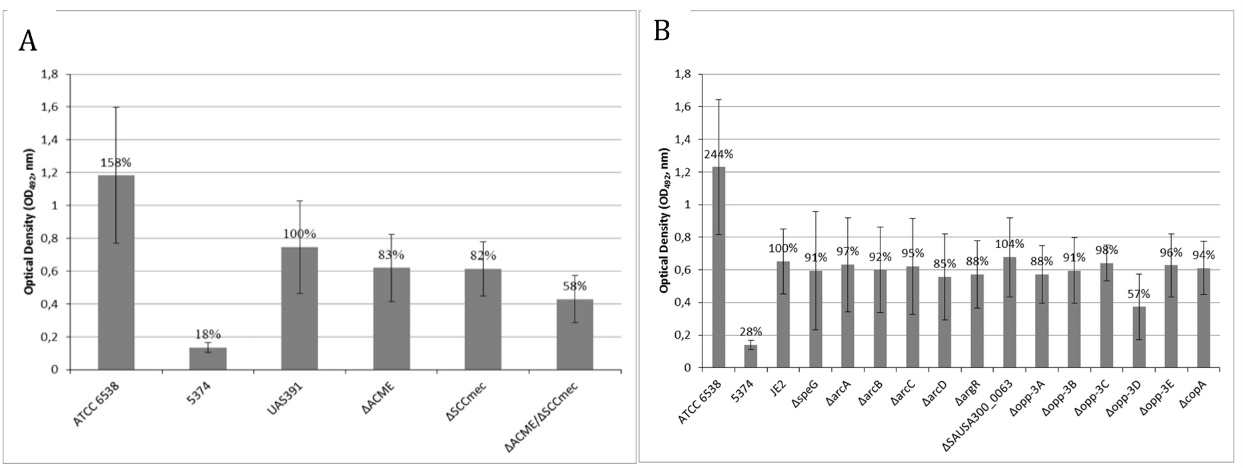


**Supplementary Figure 8.** **(A)** Formation of biofilms by UAS391 and corresponding deletion mutants of ACME and/or SCCmec mobile genetic elements under static conditions. **(B)** Formation of biofilms by USA300-JE2 and corresponding transposon insertion mutants of genes encoded by the ACME mobile genetic element under static conditions. *arcA*: arginine deiminase. *arcB*: ornithine carbamoyltransferase. *arcC*: carbamate kinase. *arcD*: arginine/ornithine antiporter. *arcR*: positive transcriptional regulator. *SAUSA300_0063*: cyclic nucleotide-binding domain protein. *opp3A*: peptide ABC transporter, peptide-binding protein. *opp3B*: oligopeptide permease, channel-forming protein. *opp3C*: oligopeptide permease, channel-forming protein. *opp3D*: ABC transporter ATP-binding protein. *opp3E*: ABC transporter ATP-binding protein. *copA*: ATPase copper transport protein. Error bars represent 95% confidence intervals. ATCC6538 and 5374 were included as positive and negative control respectively.

## Supplementary Tables

**Supplementary Table 1.** *Bursa aurealis* transposon insertion mutants in USA300-JE2 and other *S.* *aureus* knock-out isolates used in this study.

| **Name** | **Description** | **Reference** |
| --- | --- | --- |
| ΔACME | ACME element (31 kb) deletion in strain USA300 UAS391 | This study |
| ΔSCC*mec* | *SCCmec*-IV element (24 kb) deletion in strain USA300 UAS391 | This study |
| ΔACME/ΔSCC*mec* | ACME and *SCCmec*–IV element deletion in strain USA300 UAS391 | This study |
| *speG (*NR-48388*)* | JE2 Tn mutant (insertion position: 63351) in spermidine N(1)-acetyltransferase (*speG*, SAUSA300_0053; 63100..63597) | NARSA repository |
| *arcA* ACME (NR-48136) | JE2 Tn mutant (insertion position: 73785) in arginine deiminase (*arcA*, SAUSA300_0065; 73113..74348) | NARSA repository |
| *arcB* ACME (NR-47590) | JE2 Tn mutant (insertion position: 70528) in ornithine carbamoyltransferase (*arcR*, SAUSA300_0062; 69839..70837) | NARSA repository |
| *arcC* ACME (NR-47054) | JE2 Tn mutant (insertion position: 69656) in carbamate kinase (*arcC*, SAUSA300_0061; 68890..69819) | NARSA repository |
| *arcD* ACME (NR-47668) | JE2 Tn mutant (insertion position: 72892) in arginine/ornithine antiporter (*arcD*, SAUSA300_0064; 71606..73027) | NARSA repository |
| *argR* ACME (NR-47776) | JE2 Tn mutant (insertion position: 74980) in arginine repressor (*argR*, SAUSA300_0066; 74617..75063) | NARSA repository |
| SAUSA300_0063 (NR-46840) | JE2 Tn mutant (insertion position: 71563) in cyclic nucleotide-binding domain protein (SAUSA300_0063; 70875..71564) | NARSA repository |
| *opp3A* (NR-46770) | JE2 Tn mutant (insertion position: 80584) in peptide ABC transporter, peptide-binding protein (*opp3A*, SAUSA300_0073; 80394..81947) | NARSA repository |
| *opp3B* (NR-48041) | JE2 Tn mutant (insertion position: 82421) in oligopeptide permease, channel-forming protein (*opp3B*, SAUSA300_0074; 81950..82906) | NARSA repository |
| *opp3C* (NR-47413) | JE2 Tn mutant (insertion position: 83147) in oligopeptide permease, channel-forming protein (*opp3C*, SAUSA300_0075; 82906..83673) | NARSA repository |
| *opp3D* (NR-48172) | JE2 Tn mutant (insertion position: 83911) in ABC transporter ATP-binding protein (*opp3D*, SAUSA300_0076; 83640..84407) | NARSA repository |
| *opp3E* (NR-48145) | JE2 Tn mutant (insertion position: 84977) in ABC transporter ATP-binding protein (*opp3E*, SAUSA300_0077; 84400..85035) | NARSA repository |
| *copA* (NR-47133) | JE2 Tn mutant (insertion position: 86401) in ATPase copper transport protein (*copA*, SAUSA300_0078; 86094..88118) | NARSA repository |
| *arcA* (NR-47166) | JE2 Tn mutant (insertion position: 2782592) in arginine deiminase (*arcA*, SAUSA300_2570; 2781714..2782949) | NARSA repository |
| *arcB* (NR-48009) | JE2 Tn mutant (insertion position: 2781207) in ornithine carbamoyltransferase (*arcB*, SAUSA300_2569; 2780671..2781681) | NARSA repository |
| *arcC_1* (NR-48261) | JE2 Tn mutant (insertion position: 1163307) in carbamate kinase (*arcC*, SAUSA300_1063;1162602..1163534) | NARSA repository |
| *arcC_2* (NR-48206) | JE2 Tn mutant (insertion position: 2778632) in carbamate kinase (*arcC*, SAUSA300_2567; 2778192..2779133) | NARSA repository |
| *arcD* (NR-46645) | JE2 Tn mutant (insertion position: 2780356) in arginine/ornithine antiporter (*arcD*, SAUSA300_2568: 2779150..2780580) | NARSA repository |
| *arcR* (NR-46997) | JE2 Tn mutant (insertion position: 2778015) in transcriptional regulator, Crp/Fnr family (*arcR*, SAUSA300_2566; 2777389..2778093) | NARSA repository |

**Supplementary Table 2.** Gene-specific primers used for RT-PCR validation of RNA-Seq results. GyrB-primers were taken from Sihto et al. (2014), while all other primers were developed in-house.

| **Gene** | **Forward primer** | **Sequence (5’ 3’)** | **Reverse primer** | **Sequence (5’ 3’)** | **Amplicon size (bp)** |
| --- | --- | --- | --- | --- | --- |
| *gyrB* | GyrB-F | GTAACACGTCGTAAATCAGCG | GyrB-R | CGTAATGGTAAAATCGCCTGC | 170 |
| *arcR* | MOarcR-F | TTTTCGCCACAAGAAAGTAGCA | MOarcR-R | ACAATCGGTTAATGCTGTGC | 180 |
|  | EMarcR-F | TTTCGCCACAAGAAAGTAGCA | EMarcR-R | AGGCCATCAATTCTCTAGGCA | 213 |
| *spxA* | MOspxA-F | TGAGGACGAGATTCGACGTTT | MOspxA-R | GTCAACCATACGTTGTGCTTCT | 76 |
| *pflA* | MOpflA-F | CCATGTCCTTGTGCCTGGTT | MOpflA-R | GGGCGCTTCGACATCTTCT | 175 |
| *clfA* | UAclfA-F | TGGCTTCAGTGCTTGTAGGT | UAclfA-R | TTTTGCGCCACACTCGTTTC | 223 |
| *arcC* | UAarcC-F | GTGGTGGCGGTATTCCAGTT | UAarcC-R | AACTTACCTTGTGCCGCGTA | 229 |
| *sodA* | UAsodA-F | GGTTCAGGTTGGGCTTGGTT | UAsodA-R | CCAATGTAGTCAGGGCGTTTG | 167 |
| *cspC* | EMcspC-F | CATCGAAAGAGAAGATGGTAGCG | EMcspC-R | CGATTGCTGAGAAGTGTACGA | 50 |
| *clpB* | EMclpB-F | GCAGCACTTCAATCTCGTGT | EMclpB-R | GTGCATCTTCCAACGCTTGT | 103 |

**Supplementary Table 3.** Significance test of transcriptomic profiles across successive timepoints in each of the two phenotypes for each strain. Calculations performed by Adonis function with 999 permutations in R. *: p ≤ 0.05.

| **Strain** | **Comparison** | **R^2^** | **P-value** |
| --- | --- | --- | --- |
| HEMRSA-15 | Biofilm timepoints | 0.54801 | 0.089 |
|  | Planktonic timepoints | 0.55787 | 0.112 |
| UAS391 | Biofilm timepoints | 0.69284 | 0.112 |
|  | Planktonic timepoints | 0.47876 | 0.022* |
| ST239-16 | Biofilm timepoints | 0.05388 | 0.956 |
|  | Planktonic timepoints | 0.15529 | 0.689 |

**Supplementary Table 4.** Significance test of transcriptomic profiles across phenotypes and successional timepoints for each strain. Calculations performed by Adonis function with 999 permutations in R. *: p ≤ 0.05.

| Strain | Component | R^2^ | P-value |
| --- | --- | --- | --- |
| HEMRSA-15 | Time | 0.44011 | 0.004* |
|  | Phenotype | 0.07486 | 0.536 |
| UAS391 | Time | 0.02917 | 0.795 |
|  | Phenotype | 0.20467 | 0.017* |
| ST239-16 | Time | 0.34865 | 0.024* |
|  | Phenotype | 0.15007 | 0.182 |

S**upplementary Table 5.** Top 20 differentially expressed genes (up and down) with |log_2_(fold change)| ≥ 1 and adjusted p-value < 0.05 in biofilms of UAS391 compared to the planktonic phenotype after 24h of growth.

| **Feature ID** | **Gene annotation** | **log_2_(fold change)** |
| --- | --- | --- |
| EX97_04255 | pathogenicity island protein | -2,59 |
| EX97_10130 | hypothetical protein | -2,55 |
| EX97_04250 | Cro/Cl family transcriptional regulator | -2,53 |
| EX97_10125 | phage head-tail adapter protein | -2,49 |
| EX97_10030 | chemotaxis protein | -2,36 |
| EX97_10135 | phage capsid protein | -2,33 |
| EX97_13300 | lactate dehydrogenase | -2,31 |
| EX97_12400 | hypothetical protein | -2,25 |
| EX97_01160 | complement inhibitor | -2,21 |
| EX97_10095 | hypothetical protein | -2,09 |
| EX97_13485 | hypothetical protein | 2,48 |
| EX97_00795 | capsular biosynthesis protein | 2,51 |
| EX97_01925 | membrane protein | 2,63 |
| EX97_13470 | arginine deiminase | 2,98 |
| EX97_02420 | veg family protein | 3,23 |
| EX97_05000 | cysteine protease | 3,98 |
| EX97_04995 | cysteine protease | 4,08 |
| EX97_13820 | cold-shock protein | 4,09 |
| EX97_05005 | glutamyl endopeptidase | 4,41 |
| EX97_04105 | cold-shock protein | 4,58 |

**Supplementary Table 6.** Top 20 differentially expressed genes (up and down) with |log_2_(fold change)| ≥ 1 and adjusted p-value < 0.05 in biofilms of HEMRSA-15 compared to the planktonic phenotype after 24h of growth.

| **Feature ID** | **Gene annotation** | **log_2_(fold change)** |
| --- | --- | --- |
| ER16_07055 | holin | -5,22 |
| ER16_07120 | tail protein | -4,83 |
| ER16_07180 | HNH endonuclease | -4,79 |
| ER16_07130 | tail protein | -4,70 |
| ER16_07155 | phage capsid protein | -4,67 |
| ER16_07160 | Clp protease ClpP | -4,61 |
| ER16_07140 | hypothetical protein | -4,59 |
| ER16_07135 | hypothetical protein | -4,53 |
| ER16_07170 | terminase | -4,50 |
| ER16_07150 | phage head-tail adapter protein | -4,41 |
| ER16_03820 | cold-shock protein | 3,59 |
| ER16_03330 | hypothetical protein | 3,65 |
| ER16_13405 | arginine deiminase | 3,66 |
| ER16_13395 | amino acid APC transporter | 3,67 |
| ER16_13385 | Crp/Fnr family transcriptional regulator | 3,68 |
| ER16_13400 | ornithine carbamoyltransferase | 3,81 |
| ER16_13390 | carbamate kinase | 3,95 |
| ER16_10640 | transglycosylase | 4,03 |
| ER16_13715 | cold-shock protein | 4,79 |
| ER16_11670 | hypothetical protein | 4,86 |

**Supplementary Table 7.** Top 20 differentially expressed genes (up and down) with |log_2_(fold change)| ≥ 1 and adjusted p-value < 0.05 in biofilms of ST239-16 compared to the planktonic bacteria after 24h of growth.

| **Feature ID** | **Gene annotation** | **log_2_(fold change)** |
| --- | --- | --- |
| L_02325 | hypothetical protein | -1,68 |
| L_01706 | hypothetical protein | -1,65 |
| L_01685 | hypothetical protein | -1,62 |
| L_02438 | hypothetical protein | -1,40 |
| L_00211 | hypothetical protein | -1,38 |
| L_00205 | hypothetical protein | -1,29 |
| L_00006 | Methicillin resistance regulatory protein MecI | -1,24 |
| L_00212 | hypothetical protein | -1,22 |
| L_00213 | hypothetical protein | -1,21 |
| L_00553 | Tyrosine recombinase XerD | -1,19 |
| L_00863 | hypothetical protein | 2,38 |
| L_02496 | ATP-dependent Clp protease ATP-binding subunit ClpL | 2,39 |
| L_02430 | hypothetical protein | 2,48 |
| L_00133 | Formate acetyltransferase | 2,56 |
| L_00718 | Fibrinogen-binding protein A | 2,66 |
| L_00134 | Pyruvate formate-lyase-activating enzyme | 2,69 |
| L_02142 | hypothetical protein | 2,91 |
| L_00729 | hypothetical protein | 3,11 |
| L_02140 | Alkaline shock protein 23 | 3,25 |
| L_00832 | Regulatory protein Spx | 3,39 |

**Supplementary Table 8.** Expression of 5 housekeeping genes (log_2_(fold change)) in the biofilm phenotype of UAS391, EMRSA-15, and ST239-16. Expression levels in the planktonic bacteria were taken as baseline 0. *gyrB*: DNA gyrase subunit B. *aroE*: shikimate dehydrogenase. *glpF*: glycerol kinase. *gmk*: guanylate kinase. *yqiL*: acetyle coenzyme A acetyltransferase.

|  | **UAS391** | | **HEMRSA-15** | | **ST239-16** | | |
| --- | --- | --- | --- | --- | --- | --- | --- |
| **Gene** | **log_2_(fold change)** | **p_adj_** | **log_2_(fold change)** | **p_adj_** | **log_2_(fold change)** | **p_adj_** |  |
| *gyrB* | -0.44 | 0.87 | 0.13 | >0.99 | 0.49 | 0.24 |  |
| *aroE* | 0.08 | 0.88 | 0.08 | >0.99 | -0.11 | 0.48 |  |
| *glpF* | 0.09 | 0.96 | 0.07 | >0.99 | -0.33 | 0.30 |  |
| *gmk* | -0.44 | 0.87 | 0.17 | >0.99 | 0.06 | 0.88 |  |
| *yqiL* | -0.16 | 0.87 | -0.57 | 0.75 | -0.26 | 0.37 |  |

**Supplementary Table 9.** Expression of 7 housekeeping genes (log_2_(fold change)) under aging biofilm growth of UAS391, HEMRSA-15, and ST239-16. Expression levels indicate fold changes in comparison to the previous timepoint. *gyrB*: DNA gyrase subunit B. *aroE*: shikimate dehydrogenase. *glpF*: glycerol kinase. *gmk*: guanylate kinase. *yqiL*: acetyl coenzyme A acetyltransferase.

| **UAS391** | | | | |
| --- | --- | --- | --- | --- |
|  | **48h** | | **72h** | |
| **Gene** | **log_2_(fold change)** | **p_adj_** | **log_2_(fold change)** | **p_adj_** |
| *gyrB* | -1.22 | 0.19 | -0.02 | >0.99 |
| *aroE* | -0.05 | 0.86 | -0.17 | 0.57 |
| *glpF* | 0.66 | 0.62 | 0.92 | 0.49 |
| *gmk* | -1.45 | 0.15 | <0.01 | >0.99 |
| *yqiL* | 0.29 | 0.43 | -0.25 | 0.47 |
| **HEMRSA-15** | | | | |
|  | **48h** | | **72h** | |
| **Gene** | **log_2_(fold change)** | **p_adj_** | **log_2_(fold change)** | **p_adj_** |
| *gyrB* | -0.87 | 0.43 | -0.24 | 0.74 |
| *aroE* | <0.01 | >0.99 | 0.13 | 0.74 |
| *glpF* | 0.45 | 0.76 | 0.60 | 0.70 |
| *gmk* | -1.66 | 0.14 | 0.55 | 0.50 |
| *yqiL* | -0.31 | 0.45 | -0.17 | 0.54 |
| **ST239-16** | | | | |
|  | **48h** | | **72h** | |
| **Gene** | **log_2_(fold change)** | **p_adj_** | **log_2_(fold change)** | **p_adj_** |
| *gyrB* | 0.55 | 0.66 | -0.66 | 0.61 |
| *aroE* | 0.25 | 0.63 | -0.02 | 0.98 |
| *glpF* | -0.11 | 0.90 | 0.57 | 0.42 |
| *gmk* | 0.97 | 0.06 | 0.56 | 0.78 |
| *yqiL* | -0.15 | 0.87 | -0.32 | 0.70 |

**Supplementary Table 10.** Topological properties of observed biofilm, planktonic, and corresponding random networks of UAS391, HEMRSA-15, and ST239-16.

|  | **HEMRSA-15** | | | | **ST239-16** | | | | **UAS391** | | | |
| --- | --- | --- | --- | --- | --- | --- | --- | --- | --- | --- | --- | --- |
|  | **Biofilm network** | **Random^a^ networks** | **Planktonic network** | **Random^a^ networks** | **Biofilm network** | **Random^a^ networks** | **Planktonic network** | **Random^a^ networks** | **Biofilm network** | **Random^a^ networks** | **Planktonic network** | **Random^a^ networks** |
| Modularity | 0.7625 | 0.2015 | 0.7963 | 0.171 | 0.9598 | 0.4142 | 0.961 | 0.4765 | 0.7679 | 0.1735 | 0.7648 | 0.1588 |
| Diameter | 2 | 4 | 2 | 3.7 | 2 | 8.343 | 1 | 9.944 | 2 | 3.822 | 2 | 3.074 |
| Density | 0.0422 | 0.0422 | 0.054 | 0.054 | 0.0148 | 0.0148 | 0.0128 | 0.0128 | 0.0531 | 0.0531 | 0.0607 | 0.0607 |
| Transity | 0.9965 | 0.0422 | 0.9993 | 0.0539 | 0.9987 | 0.0148 | 1 | 0.0126 | 0.9982 | 0.053 | 0.9976 | 0.0606 |
| Average.clustering.coefficient | 0.9904 | 0.0423 | 0.9996 | 0.0539 | 0.9984 | 0.0148 | 1 | 0.0127 | 0.9962 | 0.0531 | 0.9935 | 0.0606 |
| Average.degree | 14.1075 | 14.1075 | 18.3059 | 18.3059 | 4.3024 | 4.3024 | 3.4872 | 3.4872 | 17.9351 | 17.9351 | 20.3798 | 20.3798 |
| Edge.number | 2363 | 2363 | 3112 | 3112 | 626 | 626 | 476 | 476 | 3040 | 3040 | 3434 | 3434 |
| Node.number | 335 | 335 | 340 | 340 | 291 | 291 | 273 | 273 | 339 | 339 | 337 | 337 |
| Centralization.of.betweenness | 0.0005 | 0.0096 | 0 | 0.0071 | 0 | 0.0436 | 0 | 0.0591 | 0 | 0.0073 | 0 | 0.0063 |
| Centralization.of.eigenvector.centrality | 0.868 | 0.4881 | 0.864 | 0.4415 | 0.9689 | 0.7447 | 0.9705 | 0.793 | 0.8548 | 0.4454 | 0.8422 | 0.4232 |
| Connected.components | 52 | 1 | 46 | 1 | 72 | 4.875 | 80 | 9.358 | 46 | 1 | 43 | 1 |
| Average.path.length | 1.0304 | 2.489 | 1.0006 | 2.2984 | 1.0016 | 4.0126 | 1 | 4.5342 | 1.0026 | 2.313 | 1.0026 | 2.2125 |
| Centralization.of.closeness | 0.0006 | 0.0976 | 0.0006 | 0.1004 | 0.0001 | 0.0359 | 0.0002 | 0.0186 | 0.0007 | 0.1005 | 0.0007 | 0.0956 |
| Centralization.of.degree | 0.0925 | 0.0352 | 0.0846 | 0.0388 | 0.0196 | 0.0242 | 0.0203 | 0.0232 | 0.0949 | 0.0386 | 0.1001 | 0.041 |
| Modularity | 0.7625 | 0.2015 | 0.7963 | 0.171 | 0.9598 | 0.4142 | 0.9610 | 0.4765 | 0.7679 | 0.1735 | 0.7648 | 0.1588 |

^a^ the mean of 1000 Erdös–Réyni random networks.

**Supplementary Table 11.** Significance test of the similarity between gene expression and null model simulations along cultural succession (24h, 48h, and 72h).

| **Strain** | **Phenotype** | **Stochasticity Ratio** | **F value** | **PermANOVA p-value** | **Null Model p-value** |
| --- | --- | --- | --- | --- | --- |
| HEMRSA-15 | Biofilm | 0.1643059 | 65.172155 | 0.003 | 0.000999 |
|  | Planktonic | 0.1069279 | 50.909465 | 0.003 | 0.000999 |
| UAS391 | Biofilm | 0.1530991 | 38.842013 | 0.002 | 0.000999 |
|  | Planktonic | 0.1189083 | 55.887728 | 0.002 | 0.000999 |
| ST239-16 | Biofilm | 0.1762957 | 13.258659 | 0.002 | 0.000999 |
|  | Planktonic | 0.5446527 | 23.903645 | 0.002 | 0.000999 |
